# Crystal structure of the *Agrobacterium tumefaciens* type VI effector–immunity complex

**DOI:** 10.1107/S2053230X18016369

**Published:** 2018-11-30

**Authors:** Satoshi Fukuhara, Takanori Nakane, Keitaro Yamashita, Ryohei Ishii, Ryuichiro Ishitani, Osamu Nureki

**Affiliations:** a Department of Biological Sciences, Graduate School of Science, The University of Tokyo, 7-3-1 Hongo, Bunkyo-ku, Tokyo 113-0033, Japan

**Keywords:** *Agrobacterium tumefaciens*, type VI effector–immunity complex, crystal structure, Tae4, Tai4

## Abstract

The type VI secretion system (T6SS) comprises needle-shaped multisubunit complexes that play a role in the microbial defense systems of Gram-negative bacteria. Some Gram-negative bacteria harboring a T6SS deliver toxic effector proteins into the cytoplasm or periplasm of competing bacteria in order to lyse and kill them. To avoid self-cell disruption, these bacteria have cognate immunity proteins that inhibit their toxic effector proteins. T6SS amidase effector protein 4 (Tae4) and T6SS amidase immunity protein 4 (Tai4) are a representative of the toxic effector–immunity pairs of the T6SS. Here, the three-dimensional structures of Tai4 and the Tae4–Tai4 complex from *Agrobacterium tumefaciens* are reported at 1.55 and 1.9 Å resolution, respectively. A structural comparison with other Tae4–Tai4 homologs revealed similarities and differences in the catalytic and inhibitory mechanisms among the Tae4 and Tai4 family proteins.

## Abstract 2

The crystal structure of the type VI effector–immunity (Tae4–Tai4) complex from *Agrobacterium tumefaciens* is reported. A structural comparison with homologs revealed similarities and differences in the catalytic and inhibitory mechanisms among the Tae4 and Tai4 family proteins.

## 1. Introduction   

The type VI secretion systems (T6SSs) of Gram-negative bacteria inject various toxic effectors into the periplasmic or cytoplasmic space of the target cells and induce cell lysis of enemy cells (Hood *et al.*, 2010 ▸; MacIntyre *et al.*, 2010 ▸; Schwarz *et al.*, 2010 ▸; Murdoch *et al.*, 2011 ▸; Russell *et al.*, 2011 ▸, 2012 ▸). The various T6SS-related amidase effector proteins (Taes) are classified into four families (Tae1, Tae2, Tae3 and Tae4) based on their cleavage specificities (Russell *et al.*, 2012 ▸). These effectors and the unique bacterial secretion system, T6SS, which responds to enemy bacteria, enable Gram-negative bacteria to attack targeted heterologous cells (Russell *et al.*, 2012 ▸). In addition to these toxic effectors, Gram-negative bacteria have four amidase immunity proteins (Tai1, Tai2, Tai3 and Tai4). Tai1, Tai2, Tai3 and Tai4 neutralize the endogenous toxic effectors Tae1, Tae2, Tae3 and Tae4, respectively. These effector–immunity pairs (Tae1–Tai1, Tae2–Tai2, Tae3–Tai3 and Tae4–Tai4) generally originate from the same operons. The presence of cognate effector–immunity pairs suggests that self-protection systems with the co-expression of effector proteins and immunity proteins are a common feature in Gram-negative bacteria possessing a T6SS (Russell *et al.*, 2012 ▸).

Tae4–Tai4 is the fourth T6SS-related effector–immunity pair to be structurally determined. Previous studies reported the structures of the Tae4–Tai4 complexes from *Enterobacter cloacae*, *Salmonella typhimurium* and *Serratia marcescens* (Zhang, Gao *et al.*, 2013 ▸; Zhang, Zhang *et al.*, 2013 ▸; Benz *et al.*, 2013 ▸; Srikannathasan *et al.*, 2013 ▸). Comparisons of these structures revealed that *S. marcescens* Tai4 is structurally different from *E. cloacae* Tai4 and *S. typhimurium* Tai4, whereas the Tae4 proteins from the different species are highly conserved (Srikannathasan *et al.*, 2013 ▸). However, the crystal structures of Tae4–Tai4 complexes from other species have remained unknown.

To gain insight into the Tae4 family proteins, we determined the crystal structures of Tai4 and of the Tae4–Tai4 complex from *Agrobacterium tumefaciens* at 1.55 and 1.9 Å resolution, respectively.

## 2. Materials and methods   

### 2.1. Macromolecule production   

The genes encoding the Tai4 and Tae4 proteins from *A. tumefaciens* (ATU4346 and ATU4347, respectively) were codon-optimized for *Escherichia coli* and synthesized by Invitrogen. The *SignalP* 4.1 server (Petersen *et al.*, 2011 ▸) was used to predict the signal peptide of *A. tumefaciens* Tai4 (*At*Tai4). The *At*Tai4 gene segment (residues 26–129) without the putative signal sequence was PCR-amplified and inserted into the pCold-GST vector. The plasmid was transformed into *E. coli* Rosetta 2 (DE3) cells for protein expression.

The cells were grown in Luria–Bertani (LB) medium at 310 K until the OD_600_ reached 0.8; gene expression was then induced with 0.5 m*M* isopropyl β-d-1-thiogalactopyranoside (IPTG) following a reduction in the temperature to 277 K. Cell growth was continued for 24 h at 288 K. The N-terminally His_6_-GST-tagged *At*Tai4 was affinity-purified using an Ni–NTA column (Qiagen). The N-terminal His_6_-GST tag was removed by incubation with Turbo3C protease (Nacalai Tesque) for 16 h at 277 K. After rechromatography on the Ni–NTA column, further purification was conducted by ion-exchange chromatography on a Resource Q column (GE Healthcare) and gel-filtration chromatography on a HiLoad Superdex 75 column (GE Healthcare). The purified samples were concentrated to 8.6 mg ml^−1^ for crystallization.

For co-expression of the *At*Tae4–*At*Tai4 complex, the *At*Tai4 gene segment (residues 26–129) was cloned into the first multiple cloning site of the pETDuet-1 vector (Novagen) and the *At*Tae4 gene segment (residues 1–163) was subsequently cloned into the second multiple cloning site. A *Tobacco etch virus* (TEV) protease-recognition sequence was introduced between the His_6_ tag and the *At*Tai4 sequence by a PCR-based method. The plasmid was transformed into *E. coli* Rosetta 2 (DE3) cells for overexpression. The cells were cultured in LB medium at 310 K until the OD_600_ reached 0.8; gene expression was then induced with 0.5 m*M* IPTG following a temperature reduction to 277 K. The cells were further cultured at 291 K for 24 h. The *At*Tae4–*At*Tai4 complex was affinity-purified using an Ni–NTA column (Qiagen). The N-terminal His_6_ tag was removed by incubation with TEV protease for 24 h at 277 K. After rechromatography on the Ni–NTA column, the complex was further purified by ion-exchange chromatography on a Resource Q column and subsequent gel-filtration chromatography on a HiLoad Superdex 75 column. The purified complex was concentrated to 13 mg ml^−1^ for crystallization trials.

### 2.2. Crystallization   

Initial crystallization trials were performed at 293 K by the sitting-drop vapor-diffusion method in a 96-well crystallization plate using various commercially available screening kits. Crystallization drops were prepared by mixing 200 nl purified protein solution and 200 nl reservoir solution using a Mosquito crystallization robot (TTP Labtech). The initial crystals of *At*Tai4 were optimized at 293 K by varying the concentrations of PEG and salt in the reservoir solution using an Additive Screen kit (Hampton Research). Plate-shaped crystals of *At*Tai4 were obtained in 33% PEG 6000, 1.5 *M* lithium chloride, 100 m*M* sodium acetate. The *At*Tae4–*At*Tai4 complex formed thick plate-shaped crystals using MemGold reservoir condition E11 consisting of 35% PEG 400, 0.05 *M* Tris pH 8.5, 0.05 *M* sodium sulfate, 0.05 *M* lithium sulfate.

### 2.3. Data collection and processing   

All crystals were cryoprotected in reservoir solution supplemented with 25% ethylene glycol and flash-cooled in a nitrogen-gas stream. X-ray diffraction data for *At*Tai4 and for the *At*Tae4–*At*Tai4 complex were collected on beamlines BL41XU and BL32XU at SPring-8, Hyogo, Japan using a PILATUS3 6M detector (Dectris) and an MX225HS detector (Rayonix), respectively. The continuous helical data-collection scheme was applied using 12 × 8 µm (*At*Tai4) and 18 × 1 µm (*At*Tae4-*At*Tai4 complex) beams. Diffraction data were integrated with *DIALS* (Waterman *et al.*, 2016 ▸) and scaled with *AIMLESS* (Evans & Murshudov, 2013 ▸). The data-collection statistics are shown in Table 1 ▸.

### 2.4. Structure determination   

The structures of *At*Tai4 and the *At*Tae4–*At*Tai4 complex were solved by molecular replacement with *MOLREP* (Vagin & Teplyakov, 2010 ▸) using the structures of Tai4 from *S. marcescens* (PDB entry 3zfi; Srikannathasan *et al.*, 2013 ▸) and the Tae4–Tai4 complex from *S. marcescens* (PDB entry 4bi8; Srikannathasan *et al.*, 2013 ▸), respectively, as search models. Model building and structure refinement were performed using *Coot* (Emsley *et al.*, 2010 ▸) and *REFMAC*5 (Murshudov *et al.*, 2011 ▸), respectively. Ramachandran plot analysis was performed using *MolProbity* (Chen *et al.*, 2010 ▸). The refinement statistics are shown in Table 1 ▸. The atomic coordinates and structure factors of *At*Tai4 and the *At*Tae4–*At*Tai4 complex have been deposited in the Protein Data Bank (PDB) with accession codes 6ije and 6ijf, respectively. X-ray diffraction images have been also deposited in the Zenodo data repository (https://doi.org/10.5281/zenodo.1453302).

## 3. Results and discussion   

### 3.1. Overall structure   

**Figure 1:**
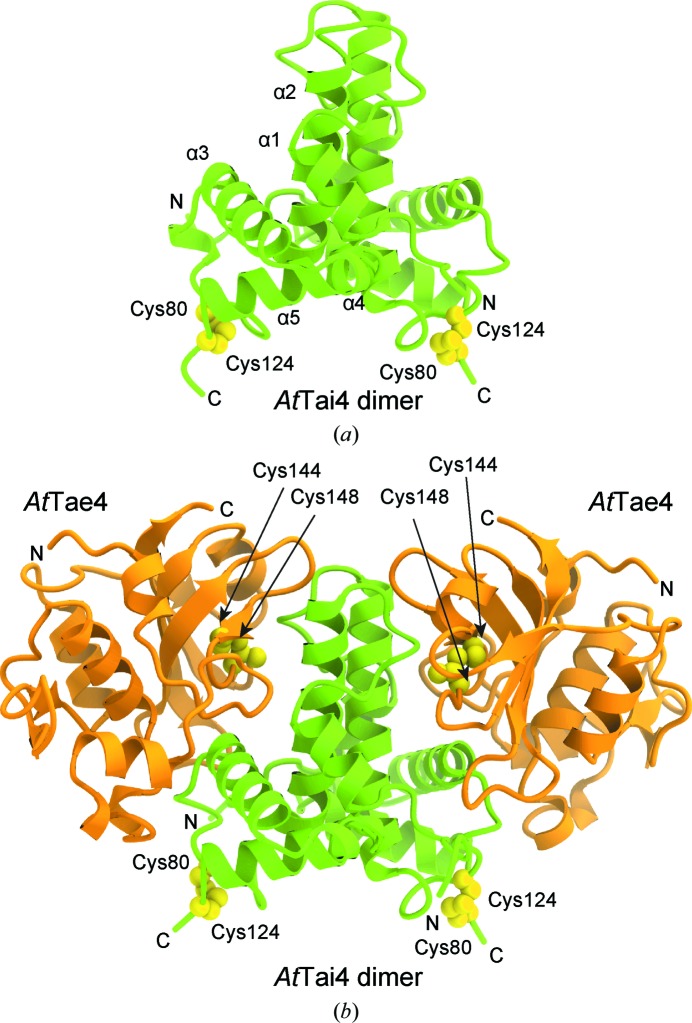
Crystal structures of the *At*Tai4 homodimer and the *At*Tae4–*At*Tai4 complex. (*a*) Structural overview of the *At*Tai4 homodimer. The intramolecular disulfide bonds between Cys80 and Cys124 are shown as yellow spheres. (*b*) Overall structure of the *At*Tae4–*At*Tai4 complex. The intramolecular disulfide bonds between Cys144 and Cys148 are shown as yellow spheres.

The crystal structure of *At*Tai4 was determined at 1.55 Å resolution. *At*Tai4 forms a homodimer composed of five α-helices (α1–α5; Fig. 1 ▸ *a*). The α2 helix (residues 52–74) contributes to dimer formation in the asymmetric unit, which is consistent with the size-exclusion chromatography results indicating that *At*Tai4 exists as a dimer in solution. A disulfide bond is formed between Cys80 and Cys124 in each protomer (Fig. 1 ▸ *a*). In addition, we determined the crystal structure of the *At*Tae4–*At*Tai4 complex at 1.9 Å resolution (Fig. 1 ▸ *b*). The structure revealed that the *At*Tai4 dimer binds two *At*Tae4 molecules to form a heterotetramer in the asymmetric unit, which is consistent with the size-exclusion chromatography results indicating that the *At*Tae4–*At*Tai4 complex exists in a heterotetrameric form in solution. The crystal structure revealed that *At*Tae4 forms an intramolecular disulfide bond between Cys144 and Cys148, which may confer structural stability (Fig. 1 ▸ *b*). Superimposition of *At*Tai4 alone and *At*Tai4 bound to *At*Tae4 resulted in a root-mean-square deviation (r.m.s.d.) value of 0.8 Å, indicating that no structural changes occur upon complex formation.

### 3.2. Structure comparison   

**Figure 2:**
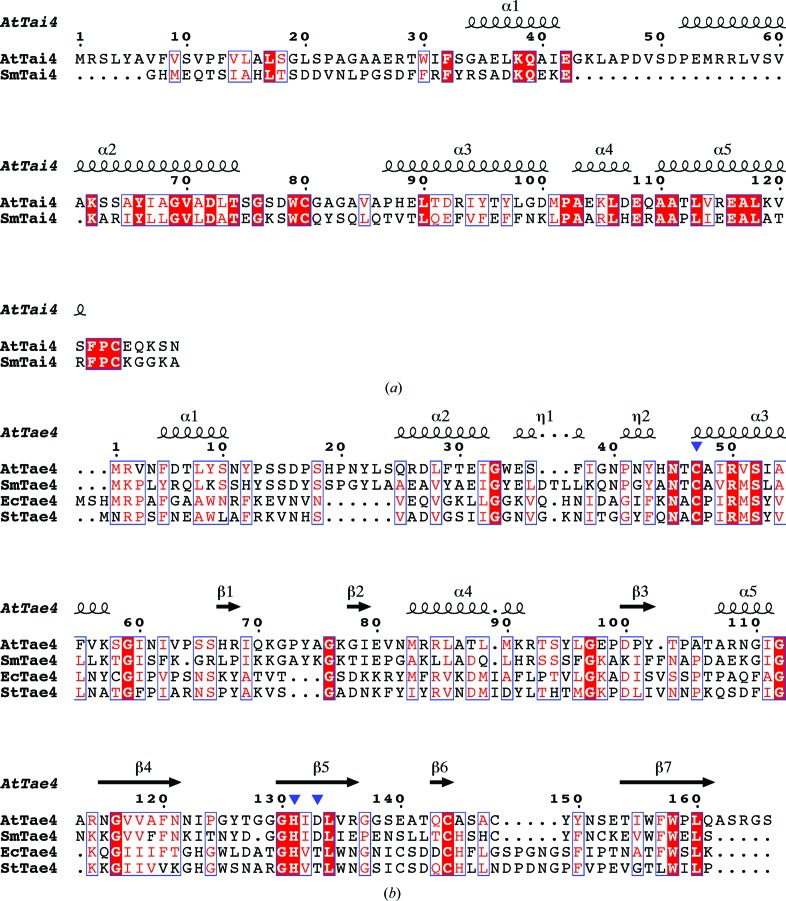
Structure-based sequence alignments of *At*Tai4 and *At*Tae4 with their homologs performed with *Clustal Omega* and *ESPript*3. (*a*) Sequence alignment of *At*Tai4 with *Sm*Tai4 (PDB entry 3zfi; Srikannathasan *et al.*, 2013 ▸). (*b*) Sequence alignment of *At*Tae4 with *Sm*Tae4 (PDB entry 4bi3; Srikannathasan *et al.*, 2013 ▸), *Ec*Tae4 (PDB entry 4hfk; Zhang, Zhang *et al.*, 2013 ▸) and *St*Tae4 (PDB entry 4j30; Benz *et al.*, 2013 ▸). The potential catalytic triad residues, Cys47, His131 and Asp133, are indicated by blue triangles.

A search for structural homologs was conducted using the *DALI* server (Holm & Laakso, 2016 ▸). The top-scoring structural homolog of *At*Tai4 was the Rap1a protein from *S. marcescens* (*Sm*Tai4; PDB entry 3zfi; Srikannathasan *et al.*, 2013 ▸), with a *Z*-score of 13.5 and an r.m.s.d. of 1.7 Å. The structural homologs of *At*Tae4 are the following proteins: the Ssp1 protein from *S. marcescens* (*Sm*Tae4; PDB entry 4bi3; Srikannathasan *et al.*, 2013 ▸), the Tae4 protein from *E. cloacae* (*Ec*Tae4; PDB entry 4hfk; Zhang, Zhang *et al.*, 2013 ▸) and the Tae4 protein from *S. typhimurium* (*St*Tae4; PDB entry 4j30; Benz *et al.*, 2013 ▸). The most similar structural homolog was the *Sm*Tae4 protein, with a *Z*-score of 25.4 and an r.m.s.d. of 1.6 Å. Amino-acid sequence alignments of *At*Tai4 and *At*Tae4 with their homologs are shown in Figs. 2 ▸(*a*) and 2 ▸(*b*). *At*Tai4 shares 32.3% amino-acid sequence identity with *Sm*Tai4. *At*Tae4 shares 41.5%, 21.9% and 20.5% sequence identity with *Sm*Tae4, *St*Tae4 and *Ec*Tae4, respectively. Therefore, the *At*Tae4–*At*Tai4 complex structure deepens our understanding of the structurally distinct interactions of the Tae4 and Tai4 family proteins.

**Figure 3:**
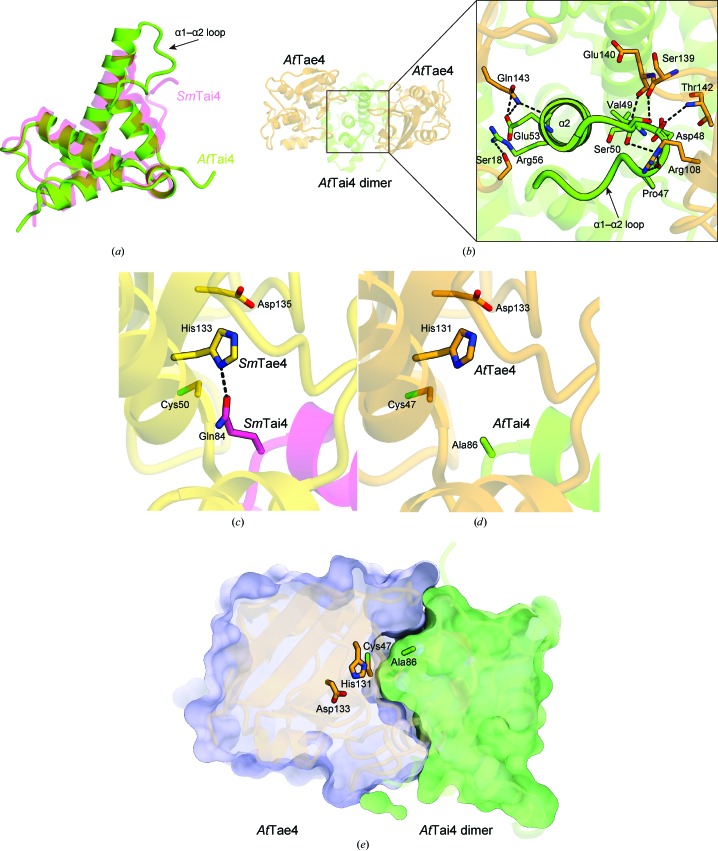
Structural comparison of the *At*Tae4–*At*Tai4 complex with the *Sm*Tae4–*Sm*Tai4 complex. (*a*) A superimposition of *At*Tai4 and *Sm*Tai4 indicated that *At*Tai4 contains extensions in the α2 helix and in the loop between the α1 and α2 helices. (*b*) The residues involved in the interaction between *At*Tai4 and *At*Tae4. (*c*) The crystal structure of the *Sm*Tae4–*Sm*Tai4 complex revealed that Gln84 of *Sm*Tai4 interacts with His133 of *Sm*Tae4 and blocks the active site (PDB entry 4bi8; Srikannathasan *et al.*, 2013 ▸). (*d*) The crystal structure of the *At*Tae4–*At*Tai4 complex lacks the interaction between the expected catalytic His131 of *At*Tae4 and the corresponding residue of *At*Tai4. The glutamine is not conserved in *At*Tai4 and is replaced by an alanine in *At*Tai4. (*e*) The *At*Tai4 homodimer is positioned close to the *At*Tae4 active-site surface and may block substrate binding.

A comparison of *At*Tai4 with *Sm*Tai4 revealed that *At*Tai4 contains a longer α2 helix and a longer loop between the α1 and α2 helices (Fig. 3 ▸ *a*). As the longer α2 helix and loop between the α1 and α2 helices interact with two *At*Tae4 molecules in the asymmetric unit (Fig. 1 ▸ *b*), *At*Tae4 and *At*Tai4 have structurally distinct interactions compared with the *Sm*Tae4–*Sm*Tai4 complex. In addition, neither the *St*Tae4–*St*Tai4 complex nor the *Ec*Tae4–*Ec*Tai4 complex has these interactions. Glu53 and Arg56 in the α2 helix form hydrogen bonds to Gln143 and Ser18 in one of the *At*Tae4 molecules in the asymmetric unit, respectively (Fig. 3 ▸ *b*). Pro47, Asp48 and Val49 in the loop of *At*Tai4 form hydrogen bonds to Arg108, Thr142 and Arg108 in the other *At*Tae4 protomer in the asymmetric unit, respectively. In addition, Ser50 in the loop of *At*Tai4 interacts with Ser139 and Glu140 in *At*Tae4 (Fig. 3 ▸ *b*). Thus, these specific interactions between *At*Tae4 and *At*Tai4 may contribute towards stabilizing the formation of the *At*Tae4–*At*Tai4 complex.

The structure of the *Sm*Tae4–*Sm*Tai4 complex revealed that *Sm*Tai4 is located at the entrance to the active site of *Sm*Tae4, where it blocks substrate access to the active site (Srikannathasan *et al.*, 2013 ▸). The catalytic Gln84 of *Sm*Tai4 forms a hydrogen bond to His133 of *Sm*Tae4 and blocks the active site (Fig. 3 ▸ *c*; Srikannathasan *et al.*, 2013 ▸). Amino-acid sequence alignment of *Sm*Tai4 and *At*Tai4 demonstrated that Gln84 of *Sm*Tai4 is not conserved and is replaced by Ala86 in *At*Tai4 (Fig. 2 ▸ *a*). In the present structure of the *At*Tae4–*At*Tai4 complex, although the potentially catalytic His131 residue of *At*Tae4 does not interact with any residues of *At*Tai4 (Fig. 3 ▸ *d*), *At*Tai4 blocks the entrance to the substrate-binding pocket of *At*Tae4 and prevents substrate access to the active site (Fig. 3 ▸ *e*).

Previous studies have suggested that *At*Tai4 and *Sm*Tai4 neutralize the activities of *At*Tae4 and *Sm*Tae4, respectively. The morphological abnormality mediated by *Sm*Tae4 was neutralized by *Sm*Tai4 (Srikannathasan *et al.*, 2013 ▸). The growth inhibition of *E. coli* DH10B owing to the expression of *At*Tae4 was rescued by the co-expression of *At*Tai4 (Ma *et al.*, 2014 ▸). While there is no direct interaction between the potentially catalytic His131 of *At*Tae4 and Ala86 of *At*Tai4, which corresponds to Gln84 of *Sm*Tai4, the structural comparison suggests that *At*Tai4 effectively neutralizes the activity of *At*Tae4 by blocking the entrance to its substrate-binding pocket.

### 3.3. Catalytic site   

**Figure 4:**
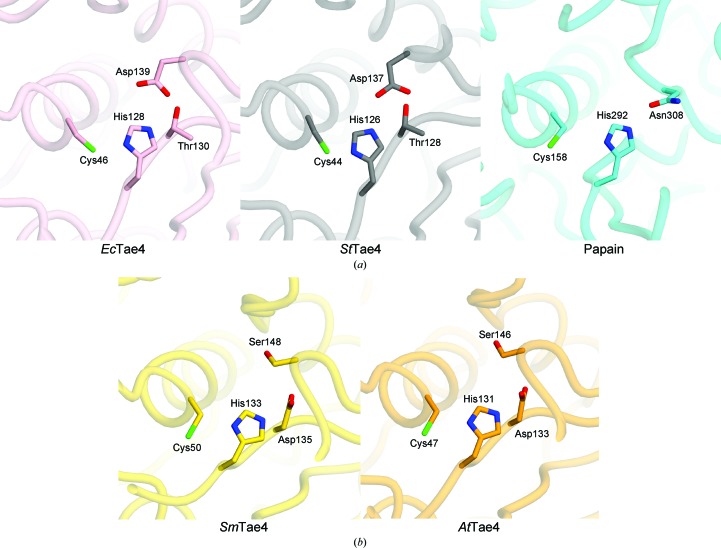
Structural differences in the catalytic triad. (*a*) *Ec*Tae4 and *St*Tae4 have a conserved catalytic active center containing the catalytic residues (Cys–His–Asp), which have a similar arrangement to the catalytic triad of papain (PDB entry 1bp4; LaLonde *et al.*, 1998 ▸). (*b*) *Sm*Tae4 and *At*Tae4 have the conserved catalytic residues (Cys–His–Asp), but the third aspartic acid in the catalytic triad has a distinct spatial arrangement.

The Tae4 family proteins have conserved catalytic residues (Cys–His–Asp) that are responsible for their peptidoglycan amidase activity. Cys46, His128 and Asp139 of *Ec*Tae4 and Cys44, His126 and Asp137 of *St*Tae4 form the catalytic triads, which are similar to the canonical catalytic triad in the papain-like cysteine peptidase (PDB entry 1bp4; LaLonde *et al.*, 1998 ▸) (Zhang, Gao *et al.*, 2013 ▸; Zhang, Zhang *et al.*, 2013 ▸; Benz *et al.*, 2013 ▸; Fig. 4 ▸ *a*). *Sm*Tae4 also has a catalytic triad formed by Cys50, His133 and Asp135. While Asp139 of *Ec*Tae4 and Asp137 of *St*Tae4 are replaced by Ser148 in *Sm*Tae4, Asp135 of *Sm*Tae4, which corresponds to Thr130 of *Ec*Tae4 and Thr128 of *St*Tae4, is located at a position similar to those of Asp139 of *Ec*Tae4 and Asp137 of *St*Tae4 in the *Sm*Tae4 structure (Figs. 4 ▸ *a* and 4 ▸ *b*). These observations suggested that Asp135 serves as the third aspartic acid residue in the catalytic triad in *Sm*Tae4 (Srikannathasan *et al.*, 2013 ▸). In the present structure of the *At*Tae4–*At*Tai4 complex, Cys47, His131 and Asp133 also form a catalytic triad, as in the *Sm*Tae4–*Sm*Tai4 complex structure (Fig. 4 ▸ *b*). Thus, the present structure re­inforces the idea that the Tae4 family proteins have two types of structurally distinct catalytic triads.

## 4. Conclusion   

In this work, we determined the crystal structures of *At*Tai4 and the *At*Tae4–*At*Tai4 complex. Comparisons of these structures with those of homologous proteins revealed that the *At*Tae4–*At*Tai4 complex shares structural similarity with the *Sm*Tae4–*Sm*Tai4 complex. A structural comparison of *At*Tai4 with *Sm*Tai4 showed that *At*Tai4 contains more extended helices and loops, which may enforce the interaction between *At*Tai4 and the adjacent *At*Tae4. A structural superimposition highlighted the differences in the spatial arrangement of the aspartic acid residue in the catalytic triad (Cys–His–Asp) among the Tae4 family proteins. The present structures enhance our understanding of the catalytic and inhibitory mechanisms of the Tae4 and Tai4 family proteins.

## Supplementary Material

PDB reference: Tai4 dimer, 6ije

PDB reference: Tae4–Tai4 complex, 6ijf

X-ray diffraction images URL: https://doi.org/10.5281/zenodo.1453302

## Figures and Tables

**Table 1:** Data-collection and refinement statistics Values in parentheses are for the outer shell.

	*At*Tai4	*At*Tae4–*At*Tai4 complex
Data collection		
Beamline	BL41XU, SPring-8	BL32XU, SPring-8
Wavelength (Å)	1.0000	1.0000
Crystal-to-detector distance (mm)	300	200
Rotation range per image (°)	0.5	0.5
Exposure time per image (s)	0.5	1.0
Oscillation range per crystal (°)	180	180
Helical translation step (µm)	0.5	0.3
No. of crystals	1	1
Space group	*P*2_1_2_1_2_1_	*P*6_1_
Unit-cell parameters (Å)	*a* = 53.92, *b* = 57.76, *c* = 71.47	*a* = *b* = 72.03, *c* = 194.35
Resolution (Å)	53.92–1.55 (1.58–1.55)	97.18–1.90 (1.94–1.90)
*R*_p.i.m._	0.029 (0.313)	0.022 (0.544)
〈*I*/σ(*I*)〉	13.0 (2.3)	15.0 (1.3)
Completeness (%)	99.5 (94.7)	99.6 (96.3)
Multiplicity	6.2 (4.2)	10.1 (7.3)
CC_1/2_	0.998 (0.807)	0.999 (0.528)
Mosaicity (°)	0.12	0.24
Refinement		
Resolution (Å)	53.92–1.55	97.18–1.90
No. of reflections	33087	44759
*R*_work_/*R*_free_	0.1742/0.1975	0.1903/0.2125
No. of atoms		
Protein	1546	4050
Ligand	28	21
Solvent	141	122
Average *B* factors (Å^2^)		
Protein	26.9	51.5
Ligand	48.4	81.2
Solvent	35.2	51.3
R.m.s. deviations		
Bond lengths (Å)	0.013	0.0089
Bond angles (°)	1.79	1.57
Ramachandran plot		
Favored (%)	97.45	96.91
Allowed (%)	2.55	2.90
Outliers (%)	0	0.19
